# Supplementary material for: Causal Relationship between Aging and Anorexia Nervosa: A White-Matter-Microstructure-Mediated Mendelian Randomization Analysis
Source: Biomedicines. 2024 Aug 16;12(8):1874. doi: 10.3390/biomedicines12081874 (PMC11351342; doi:10.3390/biomedicines12081874)
Supplement: Supplementary file 1 [file biomedicines-12-01874-s001.zip › Supplementary Figure/Figure S8/Leaveoneout plot of MR analysis for TL to ubm-b-1453.pdf]

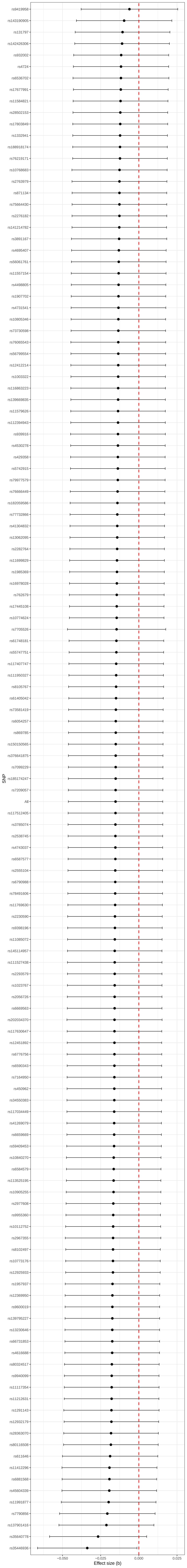

rs9419958
rs143190905
rs131797
rs142426306
rs932002
rs4724
rs6536702
rs17677991
rs11584821
rs28502153
rs17803849
rs1332941
rs188918174
rs76219171
rs10768683
rs2763979
rs871134
rs75664430
rs2276182
rs141214782
rs3891167
rs4695407
rs56061761
rs11557154
rs4498805
rs1907702
rs4731541
rs10805346
rs73730598
rs76065543
rs56799554
rs12412214
rs1003322
rs116863223
rs139669835
rs11579626
rs112394943
rs939916
rs4530278
rs429358
rs5742915
rs79977579
rs76666449
rs182059586
rs77732866
rs41304832
rs13062095
rs2282764
rs11699829
rs1985369
rs16978028
rs762679
rs17445108
rs10774624
rs7705526
rs61748181
rs55747751
rs117407747
rs111950327
rs8105767
rs61405042
rs73581419
rs6054257
rs869785
rs150150565
rs376641875
rs7099229
rs185174247
rs7209057
All
rs117512405
rs3785074
rs2538745
rs4743037
rs6587577
rs2555104
rs6790988
rs78491606
rs11769630
rs2230590
rs9398196
rs11085072
rs145114957
rs111527438
rs2293579
rs1023767
rs2056726
rs6669563
rs202034370
rs117630647
rs12451892
rs6776756
rs6590343
rs7164950
rs450962
rs34550383
rs117034449
rs41269079
rs6659669
rs59409453
rs10840270
rs6584578
rs113525195
rs10905255
rs2977608
rs9955360
rs10112752
rs2967355
rs8102497
rs10773176
rs12925933
rs1957937
rs12369950
rs9600019
rs139795227
rs13230646
rs66731853
rs4616688
rs80324517
rs9940099
rs11117354
rs11212631
rs1291143
rs12932179
rs28363070
rs80116508
rs611646
rs11412296
rs6881568
rs45604339
rs11991877
rs7790856
rs137901416
rs35640778
rs35446936
SNP
-0.050
-0.025
0.000
0.025
Effect size (b)
